# Multi-copy quantifiers for single-photon states

**DOI:** 10.1038/s41598-017-01333-y

**Published:** 2017-05-03

**Authors:** Petr Zapletal, Radim Filip

**Affiliations:** Department of Optics, Faculty of Science, Palacký University, 17, listopadu 1192/12, 771 46 Olomouc, Czech Republic

## Abstract

Single-photon states are basic resources for hybrid quantum technology with non-Gaussian states of light. Accelerating quantum technology is already able to produce high-quality single-photon states. These states can be used for hybrid quantum information processing, based on a nonclassical phase-space interference represented by negativity of a Wigner function. Therefore, new quantifiers, capable of evaluating such high-quality single-photon states, are required. We propose and analyze quantifiers which process multiple estimates of single-photon state’s statistics. The quantifiers simulate basic capability of single photons to conditionally bunch into a single mode and form a Fock state. This state exhibits complex nonclassical phase-space interference effects making its Wigner function negative in multiple areas. The quantifiers directly evaluate a presence of the multiple negativities corresponding to the Fock state. We verify applicability of the quantifiers by using them to single-photon states from recent experiments. The quantifiers can be further extended to also test indistinguishability of single-photon states. It allows to verify quantum interference of light from single-photon emitters more sensitively than in the traditional Hong-Ou-Mandel test. Besides quantum optics, the multi-copy quantifiers can be also applied to experiments with atomic memories and mechanical oscillators.

## Introduction

Development of single-photon states is crucial for a broad class of applications of quantum technology and experimental tests of principals of quantum physics. Interfering single photons are resource states required for preparation of complex quantum states of light, which are used in hybrid quantum information processing^1, 2^. The single-photon supply has already been used for conditional optical quantum operations capable of building nonlinear quantum gates for qubits^3–5^. It has also been employed in conditional noiseless quantum amplifiers^6–10^. In future, they are going to be used for simulations of highly nonlinear quantum interactions of oscillators using both conditional protocols^11–15^ and deterministic protocols^16–20^. The phase space interference of single photons is also exploited in supersensitive microscopy^21, 22^. A quality of all these applications is limited by both the statistics and indistinguishability of single photons. Ideally, single-photon states contain only one photon^23^. Two copies of the ideal single-photon state exhibit perfect Hong-Ou-Mandel interference^24^. As a result, the photons bunch to a single mode of light. In principle, it allows conditional preparation of an arbitrary *n*-photon Fock state in the single mode from *n* ideal single-photon states^25–27^, by a detection of no photons in the other modes. The Fock states are highly nonclassical states with phase-space interference effects producing a multiple negative values of the Wigner quasiprobability distribution^28^. Therefore, this bunching procedure merges negativities of Wigner functions in contrast to the previously analyzed results of conditional Gaussification of quantum states^29–31^.

Recently, a preparation of two-photon state has been demonstrated and fully tomographically analyzed by homodyne detectors^32^. An extension to *conditional preparation* of a higher Fock state |*n*〉 from *n* single-photon states has rapidly decreasing probability of success^25–27^. However, it inspires derivation of feasible and fully *deterministic quantifiers* for single-photon states. These quantifiers can calculate the output state of the bunching procedure fully deterministically. At a basic level, they evaluate data from *n* independent copies of single-photon states and deterministically determine their Fock state |*n*〉 capability. The quantifier must correspond to a physical procedure which does not increase the overall nonclassicality of the tested states. Only in this case, they can reliably evaluate the quality of the single-photon states. For this reason, the reliable quantifier testing the Fock state |*n*〉 capability corresponds to a linear optical circuit with detection of no photons in all modes except one. The Fock state |*n*〉 capability indicates that single-photon statistics is sufficiently good for a broad class of applications of single-photon states in hybrid quantum technology^1, 2^ and linear-optical discrete quantum technology^33^. The multi-copy quantifiers are not only limited to linear optical experiments, they can be applied to many hybrid discrete- and continuous-variable quantum experiments where a high-quality of single photons is required^1, 2^.

By following the physical procedure for generation of the Fock state |*n*〉 from the single photon states^25–27, 32^, a conditional output state ${\hat{\rho }}_{out}$ can be computed. This state is achievable from *n* independent statistics of single-photon states ${\hat{\rho }}_{est,i}$, $i=1,\ldots ,n$. The computed state ${\hat{\rho }}_{out}$ is used to determine a necessary condition for the Fock state |*n*〉 capability. Two kinds of multi-copy quantifiers have been previously proposed. The first kind only tests an ideal photon bunching at an unconditioned output^34^. The second kind measures fidelity of the computed conditional state ${\hat{\rho }}_{out}$ with the Fock state |*n*〉^35^. Nevertheless, none of the previous quantifiers evaluate relevant nonclassical features of the state ${\hat{\rho }}_{out}$ in phase space. This computed state ${\hat{\rho }}_{out}$ exhibits the complex nonclassical phase-space interference which is represented by multiple negative areas of a continuous-variable Wigner function^28^. Therefore, fidelity, averaging over phase-space, is not suitable. On the other hand, the negativities of the Wigner function are a very sensitive feature revealing a complexity of the phase-space interference. The negativities of the Wigner function are an important characteristic of the non-Gaussian states required for hybrid information processing^1, 2^. Moreover, this interference is required for advanced quantum computation and simulations with oscillators^16, 17^. Multiple negative areas in phase space have been recently used for very precise evaluation of non-Gaussian states in the quantum cavity electrodynamics^36^, superconducting circuits^37^, and trapped ions experiments^38^.

**Figure 1:**
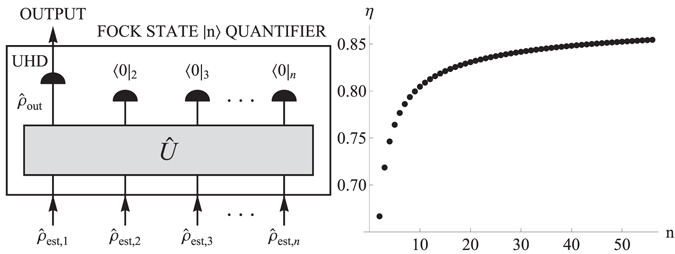
(LEFT) A pictorial representation of a physical procedure to derive the operational quantifier, processing *n* independent estimates of the density matrix ${\hat{\rho }}_{est,i}$, in terms of virtual physical operations: $\hat{U}$ - the virtual unitary transformation representing a linear optics network, 〈0|_*i*_ - the virtual detection projecting the *i*-th output mode on vacuum state, UHD - the virtual unbalanced homodyne detection measuring the point values of Wigner function of the output state ${\hat{\rho }}_{out}$ after the unitary transformation and the projections on vacuum. (RIGHT) The sufficient single-photon fraction *η* for the Fock state |*n*〉 capability of the attenuated single-photon state for which *M* = 1. Data can be fitted by the function ${\eta }_{th}(n)=0.88456-\frac{0.208243}{\sqrt{n}}-\frac{0.139454}{n}$. The largest deviation of fit function from the computed data is 9 × 10^−4^.

Here, we propose and analyze single-photon phase-space quantifier based on multiple estimates of photon statistics produced by a single-photon state. The first part of the quantifier composes of computing a conditional output Wigner function from the independent estimates of the photon statistics. The computational procedure fully corresponds to the physical conditional protocol of a Fock state preparation from single-photon states^25–27, 32^ schematically depicted in Fig. 1. In the second part, the computed output Wigner function is evaluated. In the case of ideal single-photon states, the output Wigner function fully corresponds to the Fock state |*n*〉. Negative areas of this Wigner function form concentric annuli. The number of the annuli relates to the number *n* of the photons in the Fock state. The importance of multiple Wigner function’s negativities motivates us to define the quantifier in the following manner. The number of negative annuli of the conditional output state ${\hat{\rho }}_{out}$ computed from *n* copies ${\hat{\rho }}_{est,i}$ is determined. If the number of negative annuli corresponds to the Fock state |*n*〉, the single-photon source exhibits a Fock-state |*n*〉 capability. It means that the single-photon states produced by the source can, in principle, be used to generate quantum non-Gaussian state with negativities of Wigner function corresponding to the Fock-state |*n*〉. The higher number of the concentric negative annuli in Wigner function demonstrates the higher level of the challenging continuous-variable nonclassical interference in phase space. To determine the number of negative annuli of the output Wigner function, a number of roots of a single-variable polynomial has to be only found. Coefficients of the polynomial depend on the input photon statistics. The quantifier determining the Fock state |*n*〉 capability is operational and deterministic.

First, we apply the quantifier to idealized single-photon sources without multi-photon contributions. This determines sufficient single-photon fraction to reach the Fock state |*n*〉 capability. Second, we perform a Monte-Carlo simulation with realistic photon statistics of single-photon states estimated from recent experiments^39^. The multi-photon contributions of the single-photon states and deviations in between them affect the Fock state |*n*〉 capability. The Monte-Carlo simulation demonstrates sensitivity of the quantifier to the realistic experimental imperfections. Our result offers an operational quantifier sensitively evaluating light from high-quality single-photon emitters with visible negativity of Wigner function, for which the currently used quantifiers are not sufficient. This basic level of analysis evaluates only the statistics of light from single-photon sources. It assumes an ideal interference of all states. In order to test also an ability to interfere, we propose an extension of the quantifier to an advanced level. The advanced quantifier performs the first layer of the bunching procedure, depicted in Fig. 1, in a laboratory. This quantifier then processes estimated conditional photon statistics after conditional two-copy interference experiments. As a result, we obtain the Fock state |*n*〉 capability involving jointly statistics and indistinguishability of two realistic single-photon state copies. This nonclassical interference test is extremely sensitive to statistics of photons and their indistinguishability. It is more sensitive than the commonly used Hong-Ou-Mandel interference experiment with photon counters^24^. The method can be further extended by involving more complex multi-copy interference effects^40–42^.

The proposed quantifier can be applied to all existing experiments in quantum optics capable to estimate photon-number distribution by either homodyne detection or multi-channel detectors. Moreover, the quantifiers can be applied to other physical platforms, where light is used to read out states of atoms^43^, solid-state systems^44^ and mechanical oscillators^45^.

## Results

### Processing of *n* estimated density matrices

Single-photon sources typically produce phase-insensitive states diagonal in the Fock state basis. Therefore, the multi-copy quantifier uses *n* independent copies of the most likely estimate of the phase-insensitive density matrix ${\hat{\rho }}_{est,i}={\sum }_{{m}_{i}=0\,}^{M}{p}_{{m}_{i}}{|{m}_{i}\rangle }_{i}\langle {m}_{i}|$, where |*m*_*i*_〉_*i*_ is the Fock state of the *i*-th mode, *m*_*i*_ is the number of photons in the *i*-th mode, *M* is the maximal considered photon number in all modes and $i=1,\ldots ,n$. The photon-number probabilities ${p}_{{m}_{i}}$ correspond to the *m*_*i*_-photon Fock state in the *i*-th estimated density matrix ${\hat{\rho }}_{est,i}$. The density matrices can be reliably obtained by modern quantum estimation methods based on homodyne tomography^46–49^. Alternatively, photon-number statistics can be achieved from multi-channel photon detectors^50^ and photon-number resolving detectors^51^. Although, uncertainties of the density matrix’s elements are not incorporated directly, they will be used later to investigate propagation of errors through the quantifier by the Monte-Carlo simulation, which is described in Methods. Now we derive an expression for the conditional output state density matrix. To this end, we apply the virtual physical procedure, depicted in Fig. 1, to the estimates ${\hat{\rho }}_{est,i}$. The estimate of the multi-mode state

1 $${\hat{\rho }}_{in}=\underset{i=1}{\overset{n}{\otimes }}{\hat{\rho }}_{est,i}=\underset{i=1}{\overset{n}{\otimes }}[\sum _{{m}_{i}=0}^{M}{p}_{{m}_{i}}{|{m}_{i}\rangle }_{i}\langle {m}_{i}|]=\underset{i=1}{\overset{n}{\otimes }}[\sum _{{m}_{i}=0}^{M}\frac{{p}_{{m}_{i}}}{{m}_{i}!}{({\hat{a}}_{i}^{ {\dagger } })}^{{m}_{i}}{|0\rangle }_{i}\langle 0|{({\hat{a}}_{i})}^{{m}_{i}}]$$

is the input of the quantifier procedure, where ${\hat{a}}_{i}$ and ${\hat{a}}_{i}^{ {\dagger } }$ are annihilation and creation operators of *i*-th mode. To simplify the derivation, we exchange the product and the summation of annihilation and creation operators. By multiplying the tensor product, the expression for the estimate of the overall input state can be rewritten as

2 $${\hat{\rho }}_{in}=\sum _{{m}_{1}=0}^{M}\sum _{{m}_{2}=0}^{M}\cdots \sum _{{m}_{n}=0}^{M}(\prod _{i=1}^{n}\frac{{p}_{{m}_{i}}}{{m}_{i}!})(\prod _{j=1}^{n}{({\hat{a}}_{j}^{ {\dagger } })}^{{m}_{j}})|0\rangle \langle 0|(\prod _{k=1}^{n}{({\hat{a}}_{k})}^{{m}_{k}}),$$

where

3 $$|0\rangle \langle 0|\equiv {|0\rangle }_{1}\langle 0|\otimes {|0\rangle }_{2}\langle 0|\otimes \cdots \otimes {|0\rangle }_{n}\langle 0|,$$

represents vacuum state in all modes. The estimated state ${\hat{\rho }}_{in}$ is now arranged in a form suitable for direct application of the virtual procedure depicted in Fig. 1.

The first step of the procedure corresponds to a complex interference of the input modes in a linear optics network represented by the unitary transformation $\hat{U}$. It acts on the photon operators of individual modes in the following way

4 $${\hat{a}}_{i}\to {\hat{U}}^{ {\dagger } }{\hat{a}}_{i}\hat{U}=\sum _{j=1}^{n}{U}_{i,j}{\hat{a}}_{j},{\hat{a}}_{i}^{ {\dagger } }\to \hat{U}{\hat{a}}_{i}^{ {\dagger } }{\hat{U}}^{ {\dagger } }=\sum _{j=1}^{n}{U}_{i,j}^{\ast }{\hat{a}}_{j}^{ {\dagger } },$$

where *U*_*i*,*j*_ is *n* × *n* matrix describing complex probability amplitudes of single-photon propagation through the linear network. Note, the output annihilation operators are only linear combinations of the input annihilation operators. They are not mixed with the creation operators. Therefore, the linear transformation is energy conserving. Moreover, it is a passive transformation, which cannot generate nonclassicality from classical states. It only represents a generalized rotation in overall phase space of *n* modes. Therefore, it does not increase nonclassical effects caused by the phase-space interference.

In the second step, all energy is conditionally concentrated into a single output by the virtual vacuum measurements, depicted in Fig. 1. Therefore, we swap to the Schrödinger picture to describe the measurement process. In the Schrödinger picture, the unitary transformation $\hat{U}$ changes the estimate ${\hat{\rho }}_{in}$ of the overall input state to an effective estimated state ${\hat{\rho }}_{eff}={\hat{U}}^{ {\dagger } }{\hat{\rho }}_{in}\hat{U}$. The conditional procedure concentrating light into a single mode is implemented by *n* − 1 measurements projecting the *n*-mode state ${\hat{\rho }}_{eff}$ on a vacuum states ${|0\rangle }_{2}\cdots {|0\rangle }_{n}$. The state in the output mode of the conditional procedure reads

5 $${\hat{\rho }}_{out}\propto {\langle 0|}_{2}\cdots {\langle 0|}_{n}{\hat{\rho }}_{eff}{|0\rangle }_{2}\cdots {|0\rangle }_{n}.$$

Normalization of the density matrix can be omitted, since we are going to be only interested in negative areas of Wigner function. An appearance of its negative values is independent of normalization, which only scales absolute values of Wigner function. This measurement conditionally merges all photons from input modes to the remaining output mode labeled by 1. All terms that include ${\hat{a}}_{i > 1}$ and ${\hat{a}}_{i > 1}^{ {\dagger } }$ vanish due to the vacuum projective measurement. The output state in mode 1 is proportional to the density matrix

6 $${\hat{\rho }}_{out}\propto \sum _{{m}_{1}=0}^{M}\sum _{{m}_{2}=0}^{M}\cdots \sum _{{m}_{n}=0}^{M}(\prod _{i=1}^{n}\frac{{p}_{{m}_{i}}}{{m}_{i}!})(\prod _{j=1}^{n}{({U}_{j\mathrm{,1}}^{\ast }{\hat{a}}_{1}^{ {\dagger } })}^{{m}_{j}})|0\rangle \langle 0|(\prod _{k=1}^{n}{({U}_{k\mathrm{,1}}{\hat{a}}_{1})}^{{m}_{k}}).$$

This output state can be further simplified to

7 $$\begin{array}{lll}{\hat{\rho }}_{out} & \propto  & \sum _{{m}_{1}=0}^{M}\sum _{{m}_{2}=0}^{M}\cdots \sum _{{m}_{n}=0}^{M}(\prod _{j=1}^{n}\frac{{p}_{{m}_{j}}}{{m}_{j}!}{|{U}_{j\mathrm{,1}}|}^{2{m}_{j}}){({\hat{a}}_{1}^{ {\dagger } })}^{\sum {m}_{i}}|0\rangle \langle 0|{({\hat{a}}_{1})}^{\sum {m}_{i}}\\  & = & \sum _{{m}_{1}=0}^{M}\sum _{{m}_{2}=0}^{M}\cdots \sum _{{m}_{n}=0}^{M}(\prod _{j=1}^{n}\frac{{p}_{{m}_{j}}}{{m}_{j}!}{|{U}_{j\mathrm{,1}}|}^{2{m}_{j}})(\sum {m}_{i})!|\sum {m}_{i}\rangle \langle \sum {m}_{i}|,\end{array}$$

where index $\sum {m}_{i}={\sum }_{i=1}^{n}{m}_{i}$, representing the total sum of photons, depends on the summation index *m*_*i*_. Here, we label

8 $$|\sum {m}_{i}\rangle \langle \sum {m}_{i}|\equiv {|\sum {m}_{i}\rangle }_{1}\langle \sum {m}_{i}|,$$

as there is only one output mode. Note, the factor $(\sum {m}_{i})!$ emerges from applying annihilation and creation operators to the vacuum state. All photons are merged in the single output mode. Photon statistics of the output mode strongly depends on input statistics ${p}_{{m}_{i}}$ and transition probabilities ${|{U}_{j,1}|}^{2}$. However, the output photon statistics does not depend on phases of the probability amplitudes *U*_*j*,1_. It means that no phase interference effect appears between single photons propagating from different inputs to the output. This phase insensitivity was already observed for bunching of pure single photons into a single mode^27^.

The third step includes universality of the quantifier, i.e. an independence of any prior knowledge about the estimated statistics ${p}_{{m}_{i}}$. To quantify all copies of the estimated quantum states without any preference, we use a balanced linear optics network with probabilities

9 $${|{U}_{j,1}|}^{2}=\frac{1}{n},j=1,2\ldots n.$$

By considering it, the formula for the output state density matrix simplifies to

10 $${\hat{\rho }}_{out}\propto \sum _{{m}_{1}=0}^{M}\sum _{{m}_{2}=0}^{M}\cdots \sum _{{m}_{n}=0}^{M}(\prod _{j=1}^{n}\frac{{p}_{{m}_{j}}}{{m}_{j}!})\frac{(\sum {m}_{i})!}{{n}^{\sum {m}_{i}}}|\sum {m}_{i}\rangle \langle \sum {m}_{i}|.$$

This formula can be used to calculate ${\hat{\rho }}_{out}$, respectively, its photon number distribution from *n* independent estimates of matrix elements ${p}_{{m}_{i}}$.

The fourth step is evaluation of a characteristic feature of ${\hat{\rho }}_{out}$ capable of sensitively quantifying quantum non-Gaussian states. The estimated output state ${\hat{\rho }}_{out}$ can be characterized by various nonclassical properties, which are important for advanced quantum processing^16, 17^. Currently, the highest nonclassical benchmark for non-Gaussian states is the appearance of Wigner function’s interference fringes, which lead to negative values of the Wigner function^36–38^. Therefore, the number of negative annuli of the Wigner function corresponding to the Fock state |*n*〉 is a natural and reasonable choice for the quantifier criterion. The state ${\hat{\rho }}_{out}$ has the Wigner function

11 $${W}_{out}(x,p)\propto \sum _{{m}_{1}=0}^{M}\sum _{{m}_{2}=0}^{M}\cdots \sum _{{m}_{n}=0}^{M}(\prod _{j=1}^{n}\frac{{p}_{{m}_{j}}}{{m}_{j}!})\frac{(\sum {m}_{i})!}{{n}^{\sum {m}_{i}}}{W}_{\sum {m}_{i}}(x,p),$$

where $\sum {m}_{i}={\sum }_{i=1}^{n}{m}_{i}$ and ${W}_{s}(x,p)=\frac{1}{2\pi }{(-\mathrm{1)}}^{s}\,\exp \,[-\frac{{x}^{2}+{p}^{2}}{2}]\,{L}_{s}({x}^{2}+{p}^{2})$ is the s-photon Fock-state Wigner function. The function *L*_*s*_ is the Laguerre polynomial of the order *s*. Due to the circular symmetry of the Wigner function *W*_*out*_(*x*, *p*) in phase space, the number of its negative annuli is determined by the number of roots of the polynomial *W*_*out*_(*x*, 0). This polynomial is equivalent to

12 $${P}_{n}({x}^{2})\equiv \sum _{{m}_{1}=0}^{M}\sum _{{m}_{2}=0}^{M}\cdots \sum _{{m}_{n}=0}^{M}(\prod _{j=1}^{n}\frac{{p}_{{m}_{j}}}{{m}_{j}!})\frac{(\sum {m}_{i})!(-{\mathrm{1)}}^{\sum {m}_{i}}}{{n}^{\sum {m}_{i}}}{L}_{\sum {m}_{i}}({x}^{2}).$$

Number of the polynomial’s positive roots is used as the quantifier. The quantifier polynomial (12) has a finite order in the argument *z* = *x* ^2^ > 0. The order of the polynomial is given by the maximum over all sums ${\sum }_{i=1}^{n}{m}_{i}$. This maximum is determined by *nM*, where *n* is the number of input state copies and *M* is the maximal photon number considered in the input state estimates. The operational quantifier is deterministic. However, it computes outcome of the conditional physical transformation. In the Wigner function formalism, this conditional transformation is described by a convolution of the *n* input Wigner functions *W*_*est*,*i*_(*x*, *p*) rotated in phase space by the transformation (4). The convolution is with a positive Gaussian kernel due to the projections on vacuum. Therefore, it cannot generate more negative annuli than the number *n* of the input single-photon state’s copies. The conditional process only physically combine negative values of Wigner functions in phase space.

The last, fifth step, compose of finding roots of the quantifier polynomial (12). This polynomial equation can be solved numerically or graphically, as it is demonstrated below. For *n* copies of ideal single photon with ${p}_{{m}_{i}}=1$, for all *m*_*i*_ = 1, the polynomial (12) has *n* positive roots. Therefore, the Wigner function *W*_*n*_(*x*, *p*) has *n*/2 negative concentric annuli for even *n*. For odd *n*, it has (*n* − 1)/2 negative concentric annuli and one negative disk in origin. We claim that *n* single-photon states with general ${p}_{{m}_{i}}$ have the Fock state |*n*〉 capability if numbers of negative disks and annuli correspond to the Fock state |*n*〉. A realistic single-photon state has the Fock state |*n*〉 capability only if the polynomial (12) has *n* positive roots. In the following subsections, we first apply the quantifier to idealized high-quality single-photon states. Then, in order to demonstrate practicality of the quantifier, we use it to evaluate estimates of single-photon states from recent experiments.

### Sufficient single-photon fraction for attenuated single-photon state

**Figure 2:**
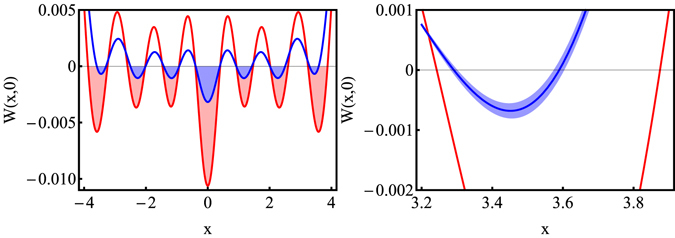
The cut through the normalized conditional output Wigner function *W*_*out*_(*x*, 0) of the virtual procedure for *n* = 7. (LEFT) The negative part of the Wigner function *W*_*out*_(*x*, 0) for the estimated realistic single-photon state ${\hat{\rho }}_{{\rm{est}}}^{\mathrm{(1)}}$ (blue region) and the negative part of the Wigner function *W*_*out*_(*x*, 0) for the attenuated single-photon state with *η* = 0.838 exhibiting the same mean photon number as ${\hat{\rho }}_{{\rm{est}}}^{\mathrm{(1)}}$ (red region). For the state ${\hat{\rho }}_{{\rm{est}}}^{\mathrm{(1)}}$, *M* = 2 and for the attenuated single photon state, *M* = 1. (RIGHT) The zoom-in to the region of the negative annulus with the largest diameter, where the blue area displays the statistical error of the Wigner function *W*_*out*_(*x*, 0) (blue line) compared to the Wigner function *W*_*out*_(*x*, 0) for the attenuated single-photon state with *η* = 0.838 (red line).

Assume *n* identical density matrices of the attenuated single-photon states ${\hat{\rho }}_{\eta }=\mathrm{(1}-\eta )|0\rangle \langle 0|+\eta |1\rangle \langle 1|$ with ${p}_{{m}_{i}=0}=\mathrm{(1}-\eta )$, ${p}_{{m}_{i}=1}=\eta$ and ${p}_{{m}_{i} > 1}=0$ for all $i=1,2\ldots n$. For these idealized states, all multi-photon contributions and any discrepancies between individual copies are neglected. Eq. (12) gives the quantifier polynomial

13 $${P}_{n}(z)\equiv \sum _{s=0}^{n}{(-1)}^{s}{\eta }^{s}{(1-\eta )}^{n-s}(\begin{array}{c}n\\ s\end{array})\frac{s!}{{n}^{s}}{L}_{s}(z),$$

where *z* > 0. Its roots can be found numerically or graphically for the given *η* and *n*. For *η* = 1, the output corresponds to the ideal Fock state |*n*〉 with $\lfloor n/2\rfloor$ negative annuli and *n* positive roots of the polynomial *P*_*n*_(*z*). For *η* < 1, Wigner function oscillations become suppressed, however, the number of negative annuli is preserved, as it is visible from Fig. 2 (LEFT). As the attenuation parameter *η* decreases, the annulus with the largest diameter narrows until the corresponding two roots of the polynomial *P*_*n*_(*z*) became equal and the negative annulus disappears. This follows from the nature of polynomial’s roots. The largest value of *η* < 1, for which two roots of *P*_*n*_(*z*) are equal, determines a threshold value. For this value of *η*, the annulus with the largest diameter vanishes. Two roots of a polynomial are equal when its discriminant Δ is equal to zero. Therefore, the threshold value of *η* is determined by the largest root of the equation

14 $${\rm{\Delta }}({P}_{n}(z\mathrm{))}\,=\,0.$$

The time demanding part of the graphical method is computation of the discriminant for the polynomial. For our purpose, computing the threshold value for up to *n* = 56 is sufficient and it was achievable in reasonable time. For *n* = 2, a sufficient condition for the Fock state |2〉 capability of single-photon state is *η* > 2/3. The sufficient *η* for the Fock state |*n*〉 capability using *n* copies of states gradually increases, as it is depicted in Fig. 1.

### n-Fock state capability for realistic single-photon states

Now, we consider a density matrices estimated from a single-photon experiment^39^. Two different density matrices are considered ${\hat{\rho }}_{{\rm{est}}}^{\mathrm{(1)}}=(0.195\pm \mathrm{0.004)}|0\rangle \langle 0|$ + $\mathrm{(0.772}\pm \mathrm{0.007)}|1\rangle \langle 1|$ + $\mathrm{(0.033}\pm \mathrm{0.003)}|2\rangle \langle 2|$ and ${\hat{\rho }}_{{\rm{est}}}^{\mathrm{(2)}}=\mathrm{(0.120}\pm \mathrm{0.007)}|0\rangle \langle 0|$ + $\mathrm{(0.857}\pm \mathrm{0.008)}|1\rangle \langle 1|$ + $\mathrm{(0.023}\pm \mathrm{0.001)}|2\rangle \langle 2|$. Monte-Carlo simulations, which are described in Methods, are used to perform the virtual procedure representing the quantifier. First, we focus on analysis of the negative values of the *mean* output Wigner function *W*_*out*_(*x*, 0) from Monte-Carlo simulations. Note, all Wigner functions presented are normalized. In Fig. 2 (LEFT), the cut through *W*_*out*_(*x*, 0) for seven input copies of ${\hat{\rho }}_{{\rm{est}}}^{\mathrm{(1)}}$ is compared to the output Wigner function for seven input copies of the attenuated single-photon state ${\hat{\rho }}_{\eta =0.838}=0.162|0\rangle \langle 0|+0.838|1\rangle \langle 1|$. The density matrix ${\hat{\rho }}_{{\rm{est}}}^{\mathrm{(1)}}$ has the same mean photon number 〈*n*〉 = 0.838 as the density matrix ${\hat{\rho }}_{\eta \mathrm{=0.838}}$ without multi-photon contributions. In the insight in Fig. 2 (RIGHT), it can be seen that the width of the negative annulus with the largest radius is distinctively smaller for the realistic single-photon state than for the attenuated single-photon state. Also the negative peaks are deeper for the attenuated single-photon state. It illustrates a destructive impact of multi-photon contributions on the Fock state |*n*〉 capability. As the graphical analysis shows, the quantifier conclusively recognizes the appearance of the negative annulus with the largest radius.

**Figure 3:**
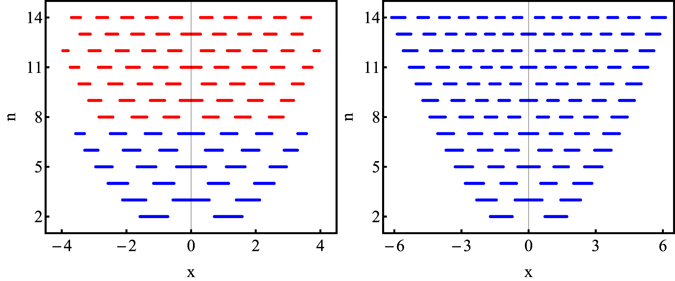
The values of the quadrature *x*, for which the cut through the output (not normalized) Wigner function *W*_*out*_(*x*, 0) exhibits negative values, plotted against the number *n* of input states of the quantifier. The inputs of the quantifier are copies of the realistic estimated density matrices ${\hat{\rho }}_{{\rm{est}}\mathrm{,1}}$ (LEFT) and ${\hat{\rho }}_{{\rm{est}}\mathrm{,2}}$ (RIGHT). For both states, *M* = 2. Blue lines denote the case when the output Wigner function exhibits the Fock state |*n*〉 like negativity. Red lines denote the case when the output Wigner function does not exhibit the full Fock state |*n*〉 like negativity.

For the realistic single-photon state ${\hat{\rho }}^{\mathrm{(1)}}$, the values of the quadrature *x*, for which the cut through the output Wigner function *W*_*out*_(*x*, 0) has negative values, is depicted against the number *n* of the input modes in Fig. 3. In the picture, one can see that the mean Wigner function *W*_*out*_(*x*, 0) exhibits only three negative annuli for eight input states. However, the Fock state |8〉 has four negative annuli. The output Wigner function still has full three negative annuli for seven input states. Therefore, the realistic state ${\hat{\rho }}_{{\rm{est}}}^{\mathrm{(1)}}$ has only the Fock state |7〉 capability. The attenuated single-photon state ${\hat{\rho }}_{\eta =0.838}$ with the same mean photon number, but without multi-photon contributions, has the Fock state |25〉 capability. This large difference arises from the small fraction of the multi-photon contribution present in realistic state ${\hat{\rho }}_{est}^{\mathrm{(1)}}$. The realistic state ${\hat{\rho }}_{{\rm{est}}}^{\mathrm{(2)}}$ with mean photon number 〈*n*〉 = 0.903 exhibits full seven negative annuli for fourteen input state copies (see Fig. 3). Therefore, the state has at least the Fock state |14〉 capability. The attenuated single-photon state with the same mean photon number, but without multi-photon contributions, exhibits an arbitrary Fock state |*n*〉 capability. It was shown up to numerical limit in the previous analysis (see Fig. 1). Our analysis is restricted by at maximum fourteen input state copies due to time limitations of our program. The limitations of the programs are discussed in Methods.

### Advanced quantifiers

**Figure 4:**
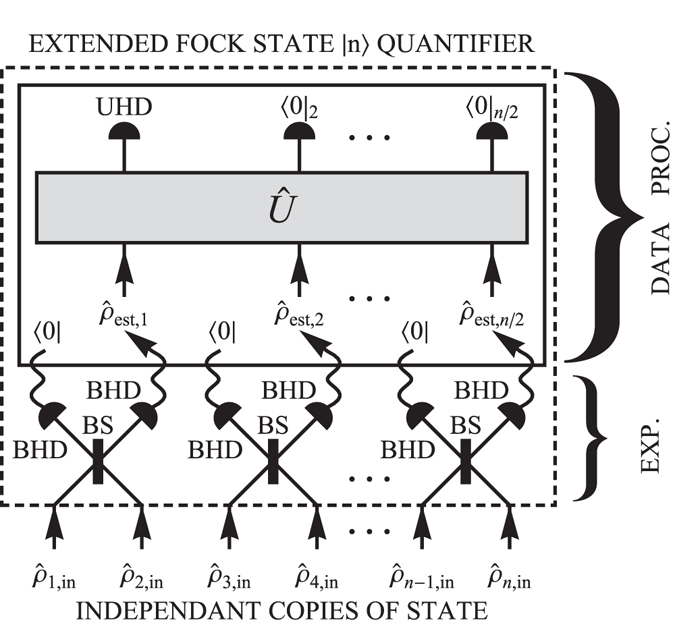
A pictorial representation of the advanced quantifier composed of the experimental part and data processing. The experimental part (EXP.): BS - the balanced beam splitter, BHD - the balanced homodyne detector. Data processing (DATA PROC.) in terms of virtual physical operations: 〈0| - the projection to vacuum (provided by a conditional data selection), ${\hat{\rho }}_{est,i}$ - the density matrices after the experimental part of the protocol estimated from data measured on BHD, $\hat{U}$ - the unitary transformation for photon creation operators representing a linear optics network, 〈0|_*i*_ - the detection projecting the *i*-th output mode to vacuum state, UHD - the unbalanced homodyne detection measuring the Wigner function of the output of the unitary procedure.

The previously introduced computational quantifier does not examine the ability of the realistic single-photon states to interfere with each other. It is only composed of data processing on the estimated statistics of individual modes. Therefore, we propose an advanced hybrid quantifier containing an experimental part implemented before the computational quantifier (see Fig. 4). The first level of the bunching procedure is performed in a laboratory. Two copies of single-photon state interfere on a balanced beam splitter. Then a projection on vacuum is conditionally induced on one mode. The tomogram of the resulting state is measured and its photon statistics is estimated. By repeating this measurement *n*/2 times, the first level of the bunching procedure is executed and *n*/2 estimated density matrices are generated. In the extension to the purely computational quantifier, the ability of single-photon pair to interfere is tested. The remaining levels of the bunching procedure are performed by computer. The estimated density matrices ${\hat{\rho }}_{{\rm{est}},{\rm{i}}}$, where $i=1,2,\ldots n/2$, are operated by data processing as in the case of the computational quantifier. As a result, the Fock state |*n*〉 capability is determined. The advanced quantifier tests a quality of the single-photon state beyond the examination provided by the purely computational quantifier. The ability to interfere is additionally tested in the experimental part of the hybrid quantifier. Recently, important experimental tests of a two-photon interference with homodyne detectors^32, 52^ have paved a way for such advanced quantifiers. The next step is an extension to quantifiers employing a three- and a four-photon interference. The extension for challenging phase-space interference tests can be straightforwardly implemented by following the presented methodology. It brings a possibility to build a hierarchy of very sensitive criteria for single-photon sources. From our expertise^53^, they will be much more sensitive than the currently used Hong-Ou-Mandel type of criteria with photon detectors.

### Outlook

We have presented new approach to evaluate high-quality single-photon sources by using multi-copy quantifiers. Principles of the methodology were demonstrated on the idealized case of attenuated single-photon states. Moreover, we used this approach to evaluate realistic single-photon statistics estimated from recent experiment.

The theoretical analysis of the concept of the Fock state |*n*〉 capability can be further extended. The multi-photon contributions have crucial effects on the Fock state |*n*〉 capability as it was demonstrated in this manuscript. In practice, the multi-photon contributions are typically small and it is hard to theoretically model them. However, they crucially reduce the Fock state capability. Therefore, further theoretical investigation depends on the experimental tests of the quantifier. The experimental verification will provide information about the predicted influence of multi-photon contributions, a stability of the single-photon generation and a quality of detection on the proposed quantifier. The Fock state |*n*〉 capability of single-photon states will depend on a quality of the density matrix estimation. Furthermore, an ability to recognize the regular phase-space interference with minima of the Wigner function reaching negative values will be crucial. Although absolute values of the negative peaks are small, they are sufficiently large in comparison to the largest negative peak close to origin. Therefore, it is very important to experimentally test the methodology on multiple independent *photon number distributions*. Moreover, an efficiency of the method needs to be examined in practice for different single-photon sources. All these experimental insides will focus the further theoretical development.

## Methods

### Monte-Carlo simulation of the procedure

The Monte-Carlo simulation employs the formula (11) to compute the output Wigner function from the diagonal elements of the estimated realistic density matrix. For the realistic state ${\hat{\rho }}_{{\rm{est}}}^{\mathrm{(1)}}$, the matrix elements ${p}_{{m}_{i}=0}$ and ${p}_{{m}_{i}=1}$ for $i=1,2,\ldots n$ are normally distributed numbers with mean values *p*_0_ = 0.195 and *p*_1_ = 0.772, respectively, and with standard deviations *s*_0_ = 0.04 and *s*_1_ = 0.07, respectively. The matrix elements ${p}_{{m}_{i}=2}=1-{p}_{{m}_{i}=0}-{p}_{{m}_{i}=1}$ for $i=1,2,\ldots n$ are complements to unity securing that the input states are normalized. For the realistic state ${\hat{\rho }}_{{\rm{est}}}^{\mathrm{(2)}}$, the matrix elements are generated analogously with appropriate mean values and standard deviations. The random sample of thirty output Wigner functions is computed in order to determine mean values and standard deviations of the output Wigner function *W*_*out*_(*x*, *p*). The values of the quadrature *x*, for which the cut through the mean output Wigner function *W*_*out*_(*x*, 0) exhibits negative values, are computed numerically. From the number of *x* intervals in which the cut is negative, the number of negative annuli is determined hence the circular symmetry of the Wigner function in phase space.

The computation of the output Wigner function *W*_*out*_(*x*, 0) is restricted by ability to perform the summation in the formula (11). The maximal achieved number of input copies for the quantifier is fourteen, which is sufficient to demonstrate the principals of the quantifier. The examination of the Fock state |*n*〉 capability of realistic single-photon states with mean photon number close to one is a problem for super computers. An example of such a state is the realistic single-photon state ${\hat{\rho }}_{{\rm{est}}\mathrm{,2}}$. These states exhibit capability of large Fock states. If we consider up to two-photon contributions in the input density matrices, the number of summands in the formula (11) grows like 3^*n*^ with the number of input modes *n*. Therefore, the computational time grows exponentially with *n*.
